# Estradiol enhanced neuronal plasticity and ameliorated astrogliosis in human iPSC-derived neural models

**DOI:** 10.1016/j.reth.2023.12.018

**Published:** 2024-01-12

**Authors:** Sopak Supakul, Chisato Oyama, Yuki Hatakeyama, Sumihiro Maeda, Hideyuki Okano

**Affiliations:** a Department of Physiology, Keio University School of Medicine, Tokyo, Japan; b Department of Electrical Engineering and Bioscience, School of Advanced Science and Engineering, Waseda University, Tokyo, Japan

**Keywords:** 17β-estradiol, Alzheimer's disease, Induced pluripotent stem cells (iPSCs), Neurons, Astrocytes

## Abstract

**Introduction:**

17β-Estradiol (E2) is a sex hormone that has been previously demonstrated to have neurotherapeutic effects on animal models of Alzheimer's disease (AD). However, clinical trials on E2 replacement therapy for preventing AD onset yielded inconsistent results. Therefore, it is imperative to clarify the therapeutic effects of E2 on human cells. In this study, we utilized induced pluripotent stem cells (iPSCs) derived from multiple AD donors to explore the therapeutic effects of E2 on the *in vitro* model of human cells.

**Methods:**

We conducted a systematic review and meta-analysis using a random-effects model of the previously reported AD clinical trials to summarize the effects of E2 replacement therapy on AD prevention. Subsequently, we induced iPSCs from the donors of the healthy control (1210B2 line (female) and 201B7 line (female)), the familial AD (APP V717L line (female) and APP KM670/671NL line (female)), and the sporadic AD (UCSD-SAD3.7 line (APOE ε3/ε3) (male), UCSD-SAD7D line (APOE ε3/ε4) (male), and TMGH-1 line (APOE ε3/ε3) (female)), then differentiated to neurons. In addition to the mono-culture model of the neurons, we also examined the effects of E2 on the co-culture model of neurons and astrocytes.

**Results:**

The meta-analysis of the clinical trials concluded that E2 replacement therapy reduced the risk of AD onset (OR, 0.69; 95 % confidence interval [CI], 0.53–0.91; I^2^ = 82 %). Neural models from the iPSCs of AD donors showed an increase in secreted amyloid-beta (Aβ) levels in the mono-culture model and an astrogliosis-like phenotype in the co-culture model. E2 treatment to the neuronal models derived from the iPSCs enhanced neuronal activity and increased neurite complexity. Furthermore, E2 treatment of the co-culture model ameliorated the astrogliosis-like phenotype. However, in contrast to the previous reports using mouse models, E2 treatment did not change AD pathogenesis, including Aβ secretion and phosphorylated tau (pTau) accumulation.

**Conclusion:**

E2 treatment of the human cellular model did not impact Aβ secretion and pTau accumulation, but promoted neuronal plasticity and alleviated the astrogliosis-like phenotype. The limited effects of E2 may give a clue for the mixed results of E2 clinical trials.

## Highlights

• The effect of E2 treatment on Alzheimer's disease was inconsistent between animal models and clinical trials.
• iPSC technology makes it possible to study the effects of E2 effects on human cells.
• E2 treatment increased neuronal activity and promoted neurite complexity.
• E2 treatment attenuated the astrogliosis-like phenotype in the co-culture model.
• The effects of E2 were consistent regardless of donor sex or disease status.

## Graphical abstract

**Figure:**
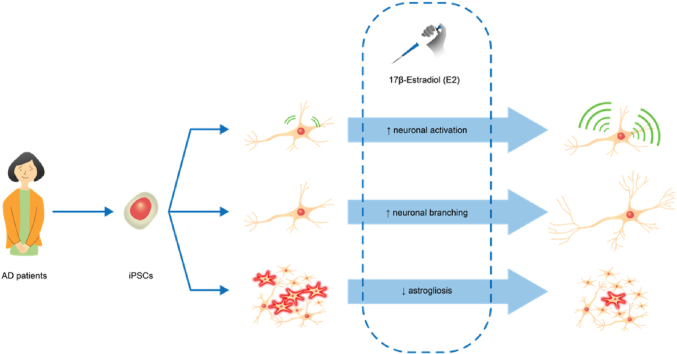

## 1 Introduction

Sex hormones play pivotal roles in the regulation of body function in addition to sex development and the reproductive system. Particularly, estrogen, a primary female steroid hormone, is recognized for its role in the regulation of the musculoskeletal system, the cardiovascular system, and the central nervous system [[1], [2], [3]]. Estrogen comprises a group of hormones: estrone (E1), estradiol (E2), and estriol (E3). Although E3 and E1 are produced in the fatty tissue, adrenal glands, or the placenta and are either biologically inactive or weakly active, E2 stands out as the most biologically active estrogen variant produced in the ovaries during a woman's reproductive years [4]. A decline in E2 levels in post-menopause women has been suggested to correlate with altered functions across several organs. For instance, a reduced E2 level is linked with the onset of osteoporosis. Consequently, hormone replacement therapy using E2 in postmenopausal women has been shown to be effective in preventing osteoporosis [5,6]. The higher prevalence of Alzheimer's disease (AD) in postmenopausal women suggests a potential association between the diminished E2 level and cognitive decline or disease onset [7].

Numerous studies have illuminated the diverse neuroprotective impacts of E2 on AD animal models, including enhanced neural plasticity, heightened resistance to oxidative stress, and reduced apoptosis [[8], [9], [10]]. Some research indicates that E2 treatment alleviates AD pathologies, such as the reduction of amyloid-beta (Aβ) plaques and neurofibrillary tangles in the ovariectomized AD mouse model [11]. Yet, clinical trials assessing the protective effect of estrogen hormone replacement therapy in postmenopausal AD women yielded inconsistent outcomes [12]. These discrepancies between animal models and human data inspired us to reconsider the impacts of E2 in human cellular models.

The advent of induced pluripotent stem cell (iPSC) technology has paved the way for investigating disease pathomechanisms using human cells [13,14]. As iPSCs can differentiate into various cell types, including central nervous system (CNS) neurons and glial cells — typically inaccessible to researchers — they offer a novel approach to modeling CNS diseases [15]. Earlier research has adeptly modeled both familial and sporadic AD (fAD and sAD) using iPSC-derived neurons, noting increased Aβ and pTau levels compared to controls [16,17]. In our study, leveraging both mono-culture models of cortical neurons and co-culture models of neurons and astrocytes, we successfully crafted human cellular models expressing AD phenotypes, intending to study E2 treatment effects.

## 2 Material and methods

### 2.1 Systematic review and meta-analysis of the past clinical studies

**Fig. 1:**
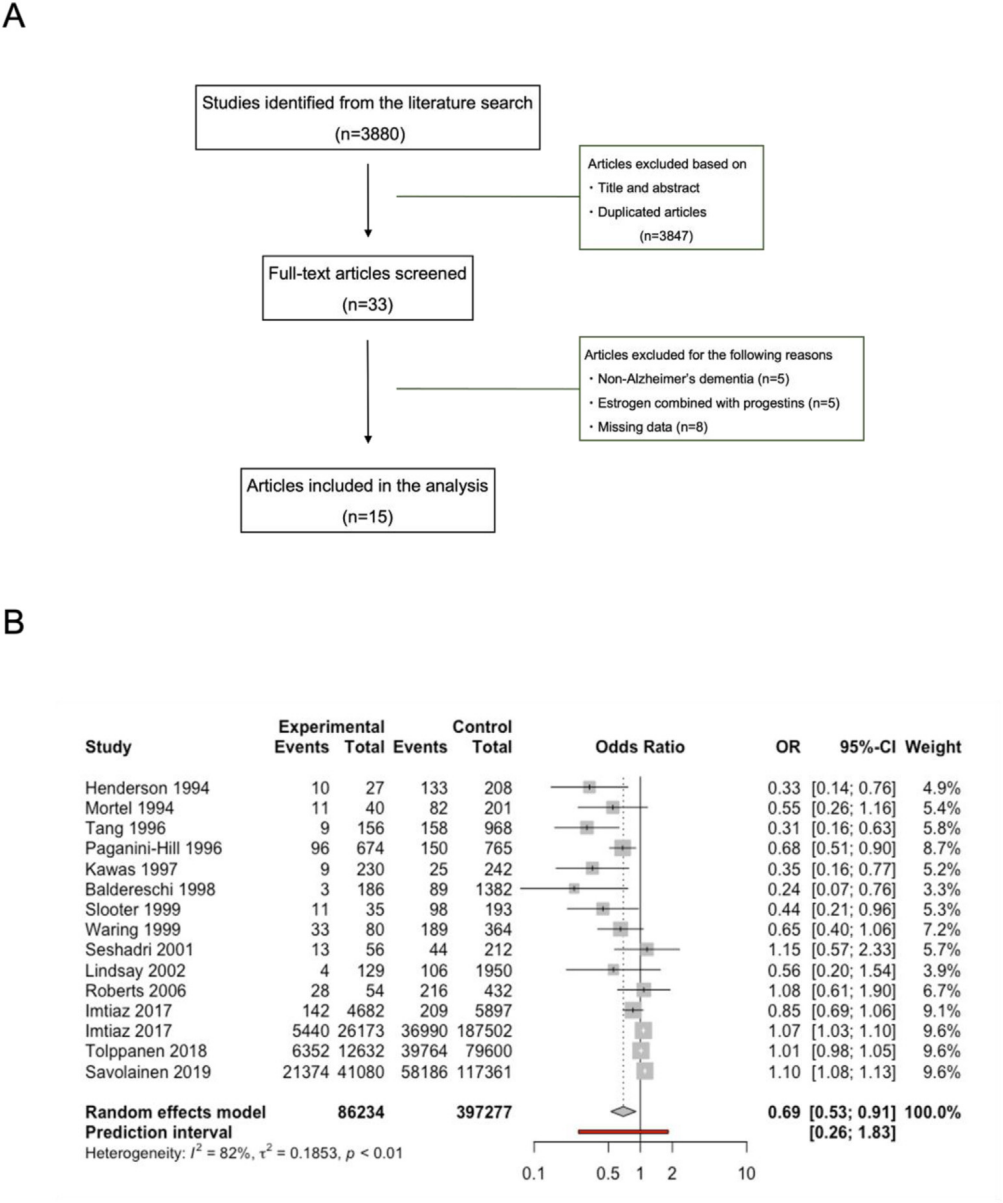
Meta-analysis of the clinical trials investigating the effects of estrogen replacement therapy and the prevalence of Alzheimer's disease. **A** Flowchart of the approach for identifying studies included in the analysis. **B** Forest plot (random-effects model) of the clinical studies included in the meta-analysis (n = 15).

A comprehensive literature search for articles related to AD onset and estrogen replacement therapy was conducted on the databases of PubMed and Web of Science. The keywords used for the literature search are (Alzheimer's OR Alzheimer OR AD OR Dementia) AND (Hormone OR Estrogen OR Oestrogen OR Risk factor) without any filters applied. A total of 3880 articles were initially identified. After screening the titles and abstracts, 3847 articles were excluded as they were not relevant to our objectives, or they were duplicated articles. After a full-text article screening of the remaining 33 articles, 18 articles were excluded as they did not contain sufficient data for our analysis. The clinical study data in the remaining 15 studies were included in the meta-analysis using a random-effects model (Fig. 1A). The results were reported in the odds ratio (OR) with 95 % confidence intervals. Details of each study included in the analysis are listed in Supplementary Table 1.

### 2.2 Human iPSC culture

The human iPSCs were cultured with StemFit AK02N medium (Ajinomoto) supplemented with 0.5 % Penicillin/Streptomycin (P/S) (Nacalai 09367-34) on the cell-culture plate coated with 3.0 μg/mL iMatrix-511 silk (Matrixome 892021). The cell culture medium was changed to a fresh AK02N medium every other day, and cells were passaged every 7 days. The cell culture followed the protocol provided by the Center for iPS Cell Research and Application (CiRA) [18]. The iPSC lines used in this study comprised the healthy control lines (1210B2 (female) and 201B7 (female)) [13,19], the familial AD lines (APP V717L (female) and APP KM670/671NL (female)) [20], and the sporadic AD lines (UCSD-SAD3.7 (APOE ε3/ε3) (male), UCSD-SAD7D (APOE ε3/ε4) (male), and TMGH-1 (APOE ε3/ε3) (female)) [21,22] (Fig. 3A).

### 2.3 Induction of neurons from the iPSCs

**Fig. 2:**
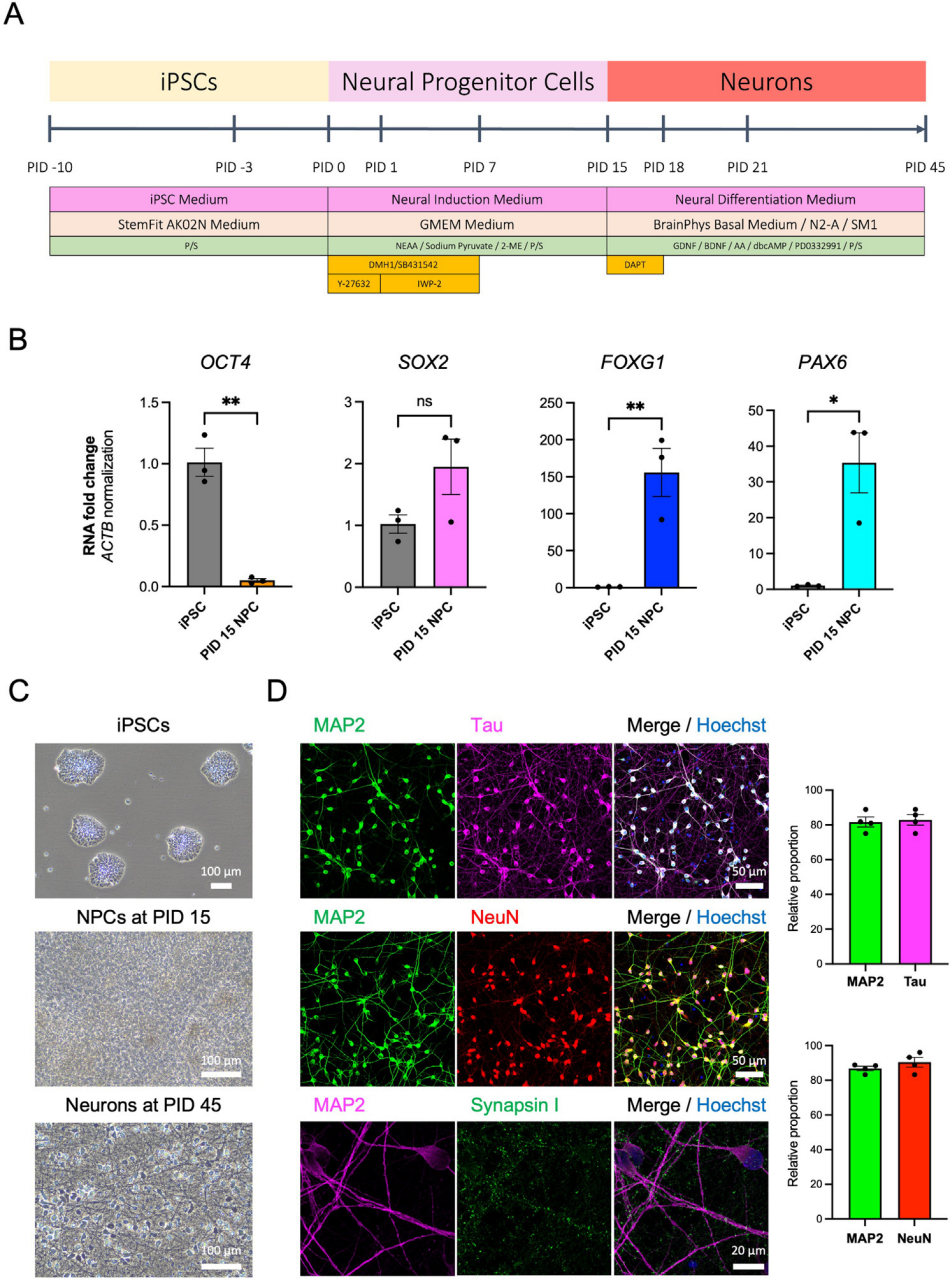
Utilizing induced pluripotent stem cells (iPSCs) as a platform to elucidate the hormonal effects in human cells. **A** Induction method for the differentiation of neurons from iPSCs using the dual SMAD inhibition. **B** RT-qPCR analysis of stem cell marker (*OCT4*), stem cell and neural progenitor cell marker (*SOX2*), and forebrain markers (*FOXG1* and *PAX6*) compared between the original iPSCs (n = 3) and the induced neural progenitor cells (NPCs) at PID 15 (n = 3). Bars, mean ± SEM. *OCT4*: *∗∗p* = 0.0011; *SOX2*: non-significant (ns); *FOXG1*: *∗∗p* = 0.0090; *PAX6*: *∗p* = 0.0151 (Unpaired *t*-test). **C** Bright-field images of the iPSCs, the induced neural progenitor cells at PID 15, and the induced neurons at PID 45 of the healthy control donor (1210B2 line). Scale bar; 100 μm. **D** Immunocytochemistry images of iPSC-derived neurons at PID 45 stained with neuronal markers (MAP2, Tau, and NeuN) and synaptic markers (Synapsin I). Scale bar; 50 μm and 20 μm. The relative proportion of markers compared to Hoechst (n = 4). Bars, mean ± SEM.

Neurons were induced from the iPSCs using dual SMAD inhibition as previously reported [23] (Fig. 2A). Before the induction of neural progenitor cells (NPCs), iPSCs were passaged onto the 6-well culture plate pre-coated with 3.0 μg/mL iMatrix-511 silk (Matrixome 892021) at 40 × 10^4^ cells per well with 1.5 mL medium supplemented with 10 μM Y-27632 (Nacalai 08945-42). The medium was changed every day with the fresh 4 mL StemFit AK02N. When the cells reached more than 80 % confluence, the medium was changed to 4 mL of the neural induction medium (GMEM medium (Wako 078-05525) supplemented with 8 % KSR (Gibco 10828010), 0.1 mM non-essential amino acid solution (NEAA) (Nacalai 06344-56), 1 mM sodium pyruvate (Nacalai 06977-34), 0.1 mM 2-mercaptoethanol (2-ME) (Gibco 21985023), and 1 % P/S (Nacalai 09367-34)) added with 2 μM DMH1 (Wako 041-33881), 2 μM SB431542 (Sigma), and 10 μM Y-27632 (Nacalai 08945-42), then started neuronal induction. On post-induction day (PID) 1–7, the medium was changed every day with fresh 4 mL of neural induction medium supplemented with 2 μM DMH1 (Wako 041-33881), 2 μM SB431542 (Sigma), and 2 μM IWP-2 (Sigma). On PID 7–15, the medium was exchanged every day with fresh neural induction medium without DMH1, SB431542, and IWP-2. On PID 15, NPCs were dissociated using Accutase (Nacalai 12679-54), and re-plated using the neural differentiation medium (BrainPhys basal medium/N2-A/SM1 kit (Stem Cell Technologies) supplemented with 10 ng/mL BDNF (R&D), 10 ng/mL GDNF (Alomone labs G-240), 200 μM L-ascorbic acid (Sigma A4544), 0.5 mM dbcAMP (Nacalai 11540-61), 2 μM PD0332991 (Sigma PZ0199-5 MG), and 0.5 % P/S (Nacalai 09367-34), 10 μM Y-27632, and 10 μM DAPT (Sigma D5942)) onto the culture plates pre-coated with 1 μg/mL Poly-L-lysine solution (PLL) (Sigma P4832) and 4 ng/mL Laminin (R&D 3400-010-01) for 2 days. On PID 18, the medium was exchanged to neural differentiation medium (BrainPhys basal medium/N2-A/SM1 kit (Stem Cell Technologies) supplemented with 10 ng/mL BDNF (R&D), 10 ng/mL GDNF (Alomone labs G-240), 200 μM L-ascorbic acid (Sigma A4544), 0.5 mM dbcAMP (Nacalai 11540-61), 2 μM PD0332991 (Sigma PZ0199-5 MG), and 0.5 % P/S (Nacalai 09367-34)) without Y-27632 and DAPT. After PID 18, half of the medium was replaced with a fresh neural differentiation medium every 3 days. The induced neurons after PID 45 were used for the analysis.

### 2.4 RNA extraction and RT-qPCR

RNA from the cultured cells was extracted using the RNeasy Mini Kit (Qiagen). cDNA was synthesized from the extracted RNA using the iScript cDNA synthesis kit (Bio-Rad). Reverse transcription-quantitative polymerase chain reaction (RT-qPCR) was performed using 4 ng/μL cDNA, TB Green II, and Rox Dye II (TaKaRa) with 20 μM of each primer. The amplification was carried out using a ViiA 7 Real-Time PCR System (Thermo Fisher Scientific 4453723) according to the manufacturer's instructions. Primers are summarized in Supplementary Table 2.

### 2.5 Immunocytochemistry (ICC)

The induced neurons at PID 45 were washed with PBS, fixed with 4 % paraformaldehyde (PFA) solution (Wako 163-20145) for 15 min at room temperature, and washed 3 times with PBS. The cells were simultaneously permeabilized and blocked with a solution of 5 % fetal bovine serum (FBS) and 0.3 % Triton X-100 in PBS and incubated with the primary antibodies overnight at 4 °C. On the next day, the cells were washed 3 times with PBS and incubated with secondary antibodies for 1 h at room temperature. After cell nuclei were stained with Hoechst 33258 (Dojindo Laboratories) diluted in PBS for 15 min, the cells were washed twice with PBS, 1 time with MilliQ water, and mounted with the PermaFlour (Thermo Fisher Scientific TA-030-FM). Images of the cells were captured using a fluorescence microscope (BZ-X810; Keyence, Osaka, Japan) and a confocal microscope (Zeiss LSM700; ZEISS Group, Oberkochen, Germany). Primary and secondary antibodies are summarized in Supplementary Table 2 with the dilution ratios.

### 2.6 Enzyme-linked immunosorbent assay (ELISA)

Conditioned media of the induced neurons at PID 45 were collected for evaluation of the secreted Aβ levels. Aβ_1-40_ and Aβ_1-42_ levels were individually measured using the ELISA kit (Wako 298-64601; Wako 296-64401). First, the Standard Solution was prepared by diluting Aβ peptides with Standard Diluent at several concentrations. Then, 100 μL of the prepared Standard Solution and the cultured media were placed into the 96w plates pre-coated with each Aβ_1-40_ and Aβ_1-42_ antibody. The plates were sealed and incubated overnight at 4 °C. On the following day, the Standard Solution and the media were removed from the wells, and the wells were washed with the Wash Solution 4–5 times. 100 μL HRP-conjugated Antibody (BC05) was then dispensed to each well. The plates were sealed and incubated at 4 °C (1 h for Aβ_1-42_ and 2 h for Aβ_1-40_). After the incubation, we removed the HRP-conjugated Antibody (BC05) and washed the wells with Wash Solution 4–5 times. Next, 100 μL TMB Solution was added into each well, then the wells were incubated in a dark environment at room temperature for 30 min. After adding 100 μL Stop Solution into each well, the absorbance at 450 nm was measured using the iMark™ Microplate Reader (Bio-Rad).

### 2.7 Ca^2+^ imaging

The induced neurons at PID 45 in the 96-well format were incubated with 100 μL neuronal differentiation medium, 2 μL of 500 μM Fluo-8 indicator (AAT Bioquest 21083), and 0.8 μL of 250 μM probenecid water (ThermoFisher Scientific P36400) at 37 °C for 15 min. The Fluo-8 signals were visualized by an Olympus IX83 microscope and analyzed with the MetaMorph® Microscopy Automation and Image Analysis Software.

### 2.8 17β-estradiol (E2) solution preparation

The differentiated neural cells were treated with 100 nM of 17β-estradiol (E2) (Sigma E8875-250MG) in the neuronal differentiation medium. Dimethyl sulfoxide (DMSO) (Sigma D5879-100ML) diluted in the neuronal differentiation medium was used as the vehicle control. 100 nM 17β-estradiol solution and the vehicle solution were newly prepared for each experiment. All experiments were performed at least three times independently.

### 2.9 Neurite complexity and neuronal spine evaluations

The induced neurons at PID 45 were transfected with the plasmid DNA of pCl-DsRed (Supplementary Fig. 2A) and β-actin-EGFP [24]. Solution 1 (50 μL Opti-MEM™ I Reduced-Serum Medium (Gibco 31985-070) and 1 μL Lipofectamine® 3000 Reagent (ThermoFisher Scientific L3000001)) and solution 2 (50 μL Opti-MEM™ I Reduced-Serum Medium, 2 μL P3000™ Reagent (ThermoFisher Scientific L3000001), and a total of 1 μg plasmid DNA (0.5 μg pCl-DsRed and 0.5 μg β-actin-EGFP)) was mixed and incubated at room temperature for 5 min. The mixture was then added directly to the wells, and the plate was incubated at 37 °C for 1 h. After 1 h of incubation, the culture medium was replaced with the fresh neuronal differentiation medium supplemented with E2 or DMSO. Cells were fixed with 4 % PFA after 4 days of exposure to the E2 or DMSO solution. After immuno-staining of the cells by an anti-RFP antibody or anti-GFP antibody (Supplementary Table 2), cells were imaged using a fluorescence microscope (BZ-X810; Keyence, Osaka, Japan) and a confocal microscope (Zeiss LSM700; ZEISS Group, Oberkochen, Germany). Neurite complexity was analyzed by the protocol for Sholl analysis in the ImageJ software [25]. The number of synapses per unit length was analyzed using SynD software [26].

### 2.10 Western blotting

The cultured cells were lysed using RIPA buffer supplemented with 1 % protease inhibitor cocktail (Nacalai 25955-11) and 1 % phosphatase inhibitor cocktail (Nacalai 07575-51) and sonicated, followed by centrifugation at 14,000 rpm for 15 min. Protein concentration of the supernatant was measured using a BCA Protein Assay Kit (ThermoFisher Scientific 23225) following the manufacturer's protocol. The supernatants were diluted with 5x SDS-PAGE Protein Loading Buffer (Molecular Cloning) and MilliQ water and adjusted to equal protein concentration among samples. The adjusted samples were denatured by heat shocking at 70 °C for 5 min. Equal amounts of samples were loaded onto the Extra PAGE One Precast Gel (Nacalai 13062-84) and electrophoresed at 300 V of constant voltage for 35 min. The protein samples on the electrophoresed gel were transferred to PVDF Membrane (Millipore IPFL00010), which was priorly activated by methanol, using the Trans-Blot SD Semi-Dry transfer cell (Bio-RAD) at 90 mAmp for 90 min. The membrane was then washed three times with MilliQ water and stained with Ponceau S Staining Solution to confirm the successful transfer of protein. The membrane was blocked using 0.5 % skim milk solution for 30 min and washed three times with tTBS(1x) solution prepared from 0.05 %-tTBS(10x) solution (Nacalai 12749-21). Next, the membrane was incubated with the primary antibody diluted in the intercept (TBS) protein-free blocking buffers (LI-COR) at 4 °C overnight. On the next day, after washing the membrane using tTBS(1x) solution three times for 5 min each, the secondary antibody diluted in the intercept (TBS) protein-free blocking buffers (LI-COR) was incubated with the membrane for 1 h at room temperature. After the membrane was washed three times for 5 min each, the fluorescence signal on the membrane was visualized using an Odyssey CLx Imaging System (LI-COR, Nebraska, USA). Primary and secondary antibodies used in this study are summarized in Supplementary Table 2 with the dilution ratios.

### 2.11 Induction of astrocytes from the iPSCs

Astrocytes were induced from iPSCs as previously reported [27]. First, the feeder-free iPSCs were cultured with the mitomycin-C-treated SNL murine fibroblast feeder cells on the 0.1 % gelatin pre-coated culture dishes. The iPSCs were cultured using human ES medium (D-MEM/Ham's F-12 with Phenol Red, HEPES, and Sodium Pyruvate (DMEM/Ham's F-12 medium) (WAKO 042-30795) supplemented with 20 % KSR (Gibco 10828010), 1 % 200 mM L-Glutamine (Gibco), 0.8 % non-essential amino acid solution (NEAA) (Nacalai 06344-56), 0.1 mM 2-mercaptoethanol (2-ME) (Gibco 21985023), and 4 ng/mL fibroblast growth factor 2 (FGF2) (PeproTech) in a 37 °C, 3 % CO_2_ humidified incubator. On the first day of the induction, the iPSCs were dissociated from the feeder cells using a dissociation solution (0.25 % trypsin, 100 μg/mL collagenase Ⅳ (Invitrogen), 1 mM CaCl_2_, and 20 % KSR (Gibco 10828010)), and cultured in FGF2-free human ES medium supplemented with 10 μM Y-27632. On PID 1, cells were cultured using EB medium (D-MEM/Ham's F-12 medium (WAKO 042-30795) containing 5 % KSR (Gibco 10828010), 1 % L-Glutamine (Gibco), 0.8 % NEAA (Nacalai 06344-56), and 0.1 mM 2-mercaptoethanol (2-ME) (Gibco 21985023)) supplemented with 3 μM dorsomorphin (Santa Cruz Biotechnology), 3 μM SB431542 (Sigma), and 3 μM CHIR99021 (Focus Biomolecules) to enhance the differentiation towards neural lineage. On PID 4, the culture medium was changed to EB medium supplemented with 1 μM retinoic acid (RA) (Sigma R2625). On PID 7, 10, and 13, the medium was changed to EB medium supplemented with 1 μM RA (Sigma R2625) and 1 μM purmorphamine (PM) (Cayman). On PID 16, the EBs were dissociated into single cells using TrypLE Select (Gibco 12563) and cultured using the neurosphere medium (media hormone mix (MHM) supplemented with 2 % B-27 (GIBCO 17504-001), 0.8 % NEAA (Nacalai 06344-56), 1 μM PM (Cayman), 20 ng/mL FGF2 (PeproTech), and 10 ng/mL epidermal growth factor (EGF) (PeproTech)) in 37 °C, 5 % CO_2_ humidified incubator. The culture medium was changed with the fresh neurosphere medium every 4 days until PID 31 to generate the primary neurospheres. On PID 32, the primary neurospheres were dissociated and cultured in the neurosphere medium without PM supplement. The culture medium was changed with fresh medium every 4 days until PID 47 to generate the secondary neurospheres. On PID 48, the secondary neurospheres were dissociated into single cells, and the cells were plated at 10 × 10^5^ cells per well onto a 6-well plate pre-coated with 0.5 % Matrigel (Corning 354277). The cells were cultured using the astrocyte differentiation medium (MHM medium supplemented with 2 % B-27 (GIBCO 17504-001), 0.8 % NEAA (Nacalai 06344-56), 10 ng/mL brain-derived neurotrophic factor (BDNF) (Alomone labs B-250), and 10 ng/mL glial cell line-derived neurotrophic factor (GDNF) (Alomone labs G-240)) in 37 °C, 5 % CO_2_ humidified incubator. The culture medium was changed to fresh medium every 4–7 days until day 77. On PID 77, the iPSC-derived astrocytes were dissociated using Accutase (Nacalai 12679-54), and the dissociated cells were plated at a density of 1 × 10^6^ cells/dish in 100-mm dishes coated with 0.05 % Matrigel in astrocyte differentiation medium. On PID 78–97, the cells were dissociated and cultured using an astrocyte differentiation medium once a week to purify the astrocyte population. Astrocytes after PID 98 were used for the analysis.

### 2.12 Establishment of the co-culture model of neurons and astrocytes from the iPSCs

We followed the newly developed method to induce neurons and astrocytes individually from the iPSCs and co-cultured them later [28]. We removed DAPT from the neuronal differentiation medium after 18 days of neuronal induction, and the astrocytes pre-cultured for 98 days were plated into the wells that were pre-plated with neurons at the proportion of neurons to astrocytes as 8:1. The co-cultured neurons and astrocytes were maintained using the neural differentiation medium. We analyzed the co-cultured cells at 30 days after astrocytes were plated.

## 3 Results

### 3.1 Systematic review and meta-analysis of the clinical trials on the effects of estrogen replacement therapy targeting AD

The clinical trials of estrogen replacement therapy to prevent AD development in postmenopausal women resulted in various conclusions. While some clinical studies have suggested the positive effects of E2 in preventing the onset of AD and improving symptoms in patients, some studies concluded that E2 treatment has no significant effects on AD protection (Reviewed in [29]). To conclude the E2 effects in clinical studies, we conducted a systematic review and meta-analysis of 15 articles related to Alzheimer's disease onset and estrogen replacement therapy, which includes a total of 483,511 patients (Fig. 1A). The analysis showed that those who received E2 replacement therapy are less likely to develop AD after follow-ups (OR, 0.69; 95 % confidence interval [CI], 0.53–0.91; I^2^ = 82 %) (Fig. 1B). The symmetric shape of the funnel plot indicated the low publication bias of the studies included in our meta-analysis (Supplementary Fig. 1). Details of each study included in the analysis are listed in Supplementary Table 1 [[30], [31], [32], [33], [34], [35], [36], [37], [38], [39], [40], [41], [42], [43], [44]].

However, there was high heterogeneity across studies, with I^2^ = 82 %. The high heterogeneity could be due to the differences in the dose of E2, period of the treatment, targeted age population, and the AD diagnosis criteria across the studies. The significant variability was unexpected, in light of the favorable outcomes of E2 treatment in animal models. In order to reassess the potential therapeutic window of E2 across different stages of AD progression, we aimed to validate the effects of E2 on AD pathogenesis within human neural cells.

### 3.2 Human-derived iPSCs were successfully induced into cortical neurons

To evaluate E2 treatment effects in human cellular models, iPSCs were induced into cortical neurons via neural progenitor cells (NPCs) using dual SMAD inhibition that is recently reported (Fig. 2A) [23,45]. At PID 15, the iPSC-derived cells significantly decreased a stem cell marker (*OCT4*), increased an NPC marker (*SOX2*) and cortical brain markers (*PAX6* and *FOXG1*) in RT-qPCR (Fig. 2B), although the full confluency of the cell culture masked the morphology of the cells (Fig. 2C left middle). These suggested that we differentiated the iPSCs toward the direction of forebrain neurons via the NPC stage. To evaluate neuronal features of the further differentiated cells, we examined the expressions of neuronal markers by immunocytochemistry at PID 45 (Fig. 2C). The differentiated cells expressed mature neuronal markers, MAP2 and Tau in cell bodies and neurites, NeuN in the nucleus, and synaptic markers, Synapsin I, surrounding the neurites. The high proportion of these neuronal markers positive cells suggested the high purity of neurons in this culture (Fig. 2D).

**Fig. 3:**
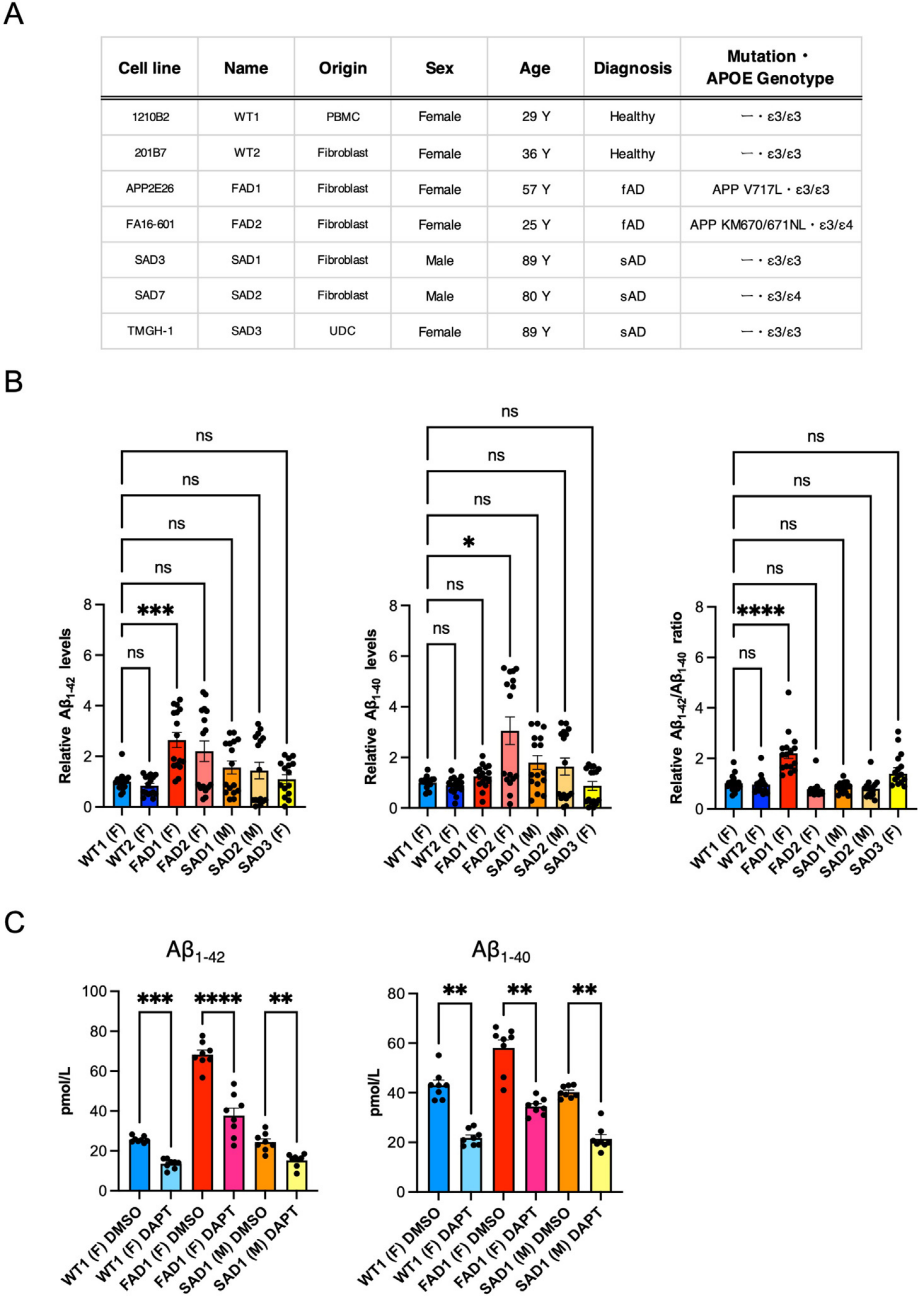
iPSCs-derived neurons from the AD patients expressed AD phenotypes. **A** Table of iPSC lines comprised of healthy control (1210B2 line (WT1) and 201B7 line (WT2)), familial AD (APP2E26 line (APP V717L) (FAD1) and FA16-601 line (APP KM670/671NL) (FAD2)), and sporadic AD (SAD3 line (APOE ε3/ε3) (SAD1), SAD7 line (APOE ε3/ε4) (SAD2), and TMGH-1 line (APOE ε3/ε3) (SAD3)) used in this study. **B** Relative Aβ_1-42_ levels, Aβ_1-40_ levels, and Aβ_1-42_/Aβ_1-40_ ratio of the iPSC-derived neurons at PID 45 (n = 16 each). Bars, mean ± SEM. Aβ_1-42_: *∗∗∗p* = 0.0009; Aβ_1-40_: *∗p* = 0.0116; Aβ_1-42_/Aβ_1-40_ ratio: ∗∗∗∗*p* < 0.0001 (Kruskal-Wallis test). **C** Aβ_1-42_ and Aβ_1-40_ levels (pmol/L) of the iPSC-derived neurons after treatment with 5 μM γ-Secretase Inhibitor (DAPT) for 2 days (n = 8 each). Bars, mean ± SEM. ∗∗*p* < 0.01; ∗∗∗*p* < 0.001; ∗∗∗∗*p* < 0.0001 (Kruskal-Wallis test).

Next, a total of 7 iPSC lines comprising the healthy control, familial AD, and sporadic AD were differentiated into neurons as described above (Fig. 3A) and analyzed at PID 45. To assess the AD phenotypes in our cellular model, we measured secreted Aβ levels in the conditioned media by ELISA. The Aβ_1-40_ levels were increased in the APP KM670/671NL line, while the ratio of Aβ_1-42_/Aβ_1-40_ was significantly increased only in the APP V717L line (Fig. 3B) like previously reported in animal models with the same mutations [[46], [47], [48]]. In addition, the treatment of 5 nM DAPT (a γ-secretase inhibitor) decreased the levels of secreted Aβ (Fig. 3C). Thus, these results demonstrated that the induced neurons from the iPSCs recapitulated the AD phenotype and responded to the drug treatment.

### 3.3 E2 treatment elevated neuronal activity, neurite branching, but not synaptic plasticity

**Fig. 4:**
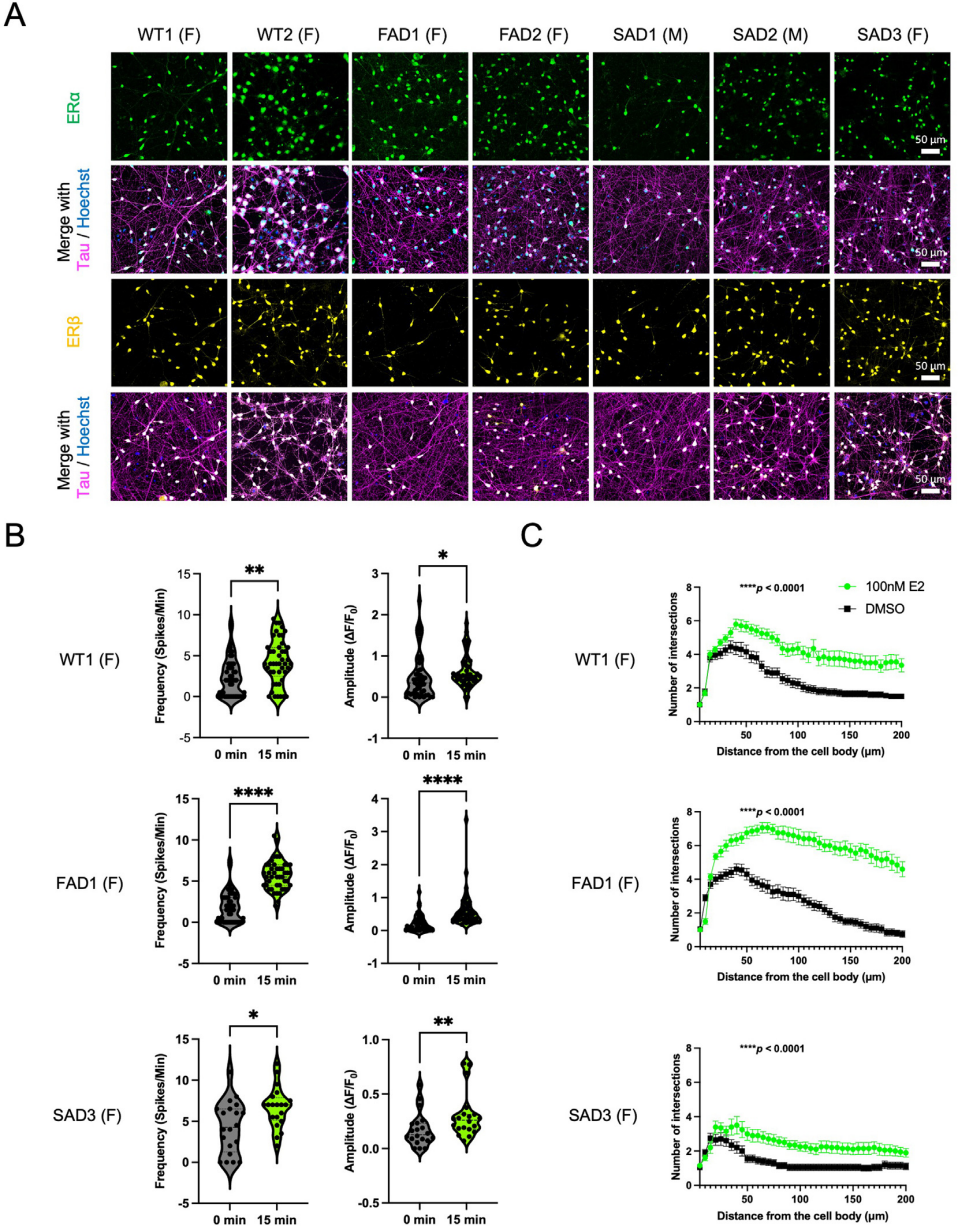
Treatment with 17β-estradiol (E2) elevated neuronal activities and neurite branching of the iPSC-derived neurons after PID 45 (line WT1, FAD1, and SAD3). **A** Immunocytochemistry images of iPSC-derived neurons at PID 45 stained with anti-estrogen receptor antibodies (ERs) (ERα or ERβ). Scale bar; 50 μm. **B** Ca^2+^ oscillations of the iPSC-derived neurons at PID 45 after 15-min 100 nM E2 treatment measured with Ca^2+^ imaging (n = 45 each). WT1: *∗∗p* = 0.0010 & *∗p* = 0.0203; FAD1: *∗∗∗∗p* < 0.0001 & *∗∗∗∗p* < 0.0001; SAD3: *∗p* = 0.0142 & *∗∗p* = 0.0087 (Mann Whitney test). **C** Increased neurite branching of iPSC-derived neurons after 4-day 100 nM E2 treatment measured by Sholl analysis (n = 20 each). WT1, FAD1, SAD3: ∗∗∗∗*p* < 0.0001 (Two-way ANOVA).

To evaluate the E2 effects in human neurons, we planned to expose the established neuronal model to E2. Before the evaluation of E2 treatment effects, we confirmed the expression of estrogen receptors including the estrogen receptor alpha (ERα) and estrogen receptor beta (ERβ) in the iPSC-derived neurons from both male and female donors (Fig. 4A). The previous study indicated that E2 elevated Ca^2+^ oscillations in the neurons differentiated from human embryonic stem cells [49]. To examine the effects of E2 in elevating Ca^2+^ oscillations of iPSC-derived neurons, we first recorded Ca^2+^ oscillations at baseline using the Fluo-8 indicator. Then, we exposed the neuron to 100 nM E2 for 15 min and recorded Ca^2+^ oscillations again at same imaging fields. The E2 treatment for short time period, 15 min, significantly increased both of the frequency (Spikes/Min) and amplitude (ΔF/F_0_) of Ca^2+^ oscillations in all neurons from both male and female donors, and non-AD and AD donors except for the frequency of FAD2 and SAD2 (Fig. 4B) (Supplementary Video 1) (Supplementary Fig. 3A). However, even for the FAD2 and SAD2 neurons, a clear trend toward an increase in frequency was observed. These results suggested that E2 acutely increases neuronal activity regardless of donor sex and disease status.

To investigate the morphological changes after the treatment with E2 including promotion of neurite branching as previously described in a single healthy male iPSC line [50], we labeled the induced neurons by introducing the pCl-dsRed plasmid to the cells. Then, the cells were treated with 100 nM E2 for 4 days. The 4-day treatment of 100 nM E2 significantly promoted the neurite branching and complexity compared to treatment with the vehicle control in all iPSC-derived neurons we tested (Fig. 4C) (Supplementary Fig. 2A and 2B) (Supplementary Fig. 3B). These results suggested that chronic treatment of E2 induced structural change in neurons regardless of sex. Therefore, both of acute and chronic effects of E2 were confirmed in iPSC lines from both male and female, and non-AD and AD donors.

To further evaluate the effects of E2 in synaptic plasticity, we examined the expression of synapsin I, a pre-synaptic protein, and dendritic spines after E2 treatment. Although the synapsin I signal was detected in the induced neurons, the synapsin I level did not differ between the E2 and vehicle-treated groups (Supplementary Fig. 4A and 4B). Next, by introducing the β-actin-EGFP DNA plasmid to the induced neurons, the dendritic spines were visualized (Supplementary Fig. 5A and 5B). However, there was no difference between the number of spines of the induced neurons even after the 4-day 100 nM E2-treatment that enhanced neurite branching as shown above (Supplementary Fig. 5C, 5D, 5E). The same results were obtained from the induced neurons of all the cell lines. Thus, the treatment with E2 did not alter synaptic plasticity at both sides of pre- and post-synaptic compartments.

### 3.4 E2 treatment did not alleviate AD-related pathogenesis in the induced neurons

**Fig. 5:**
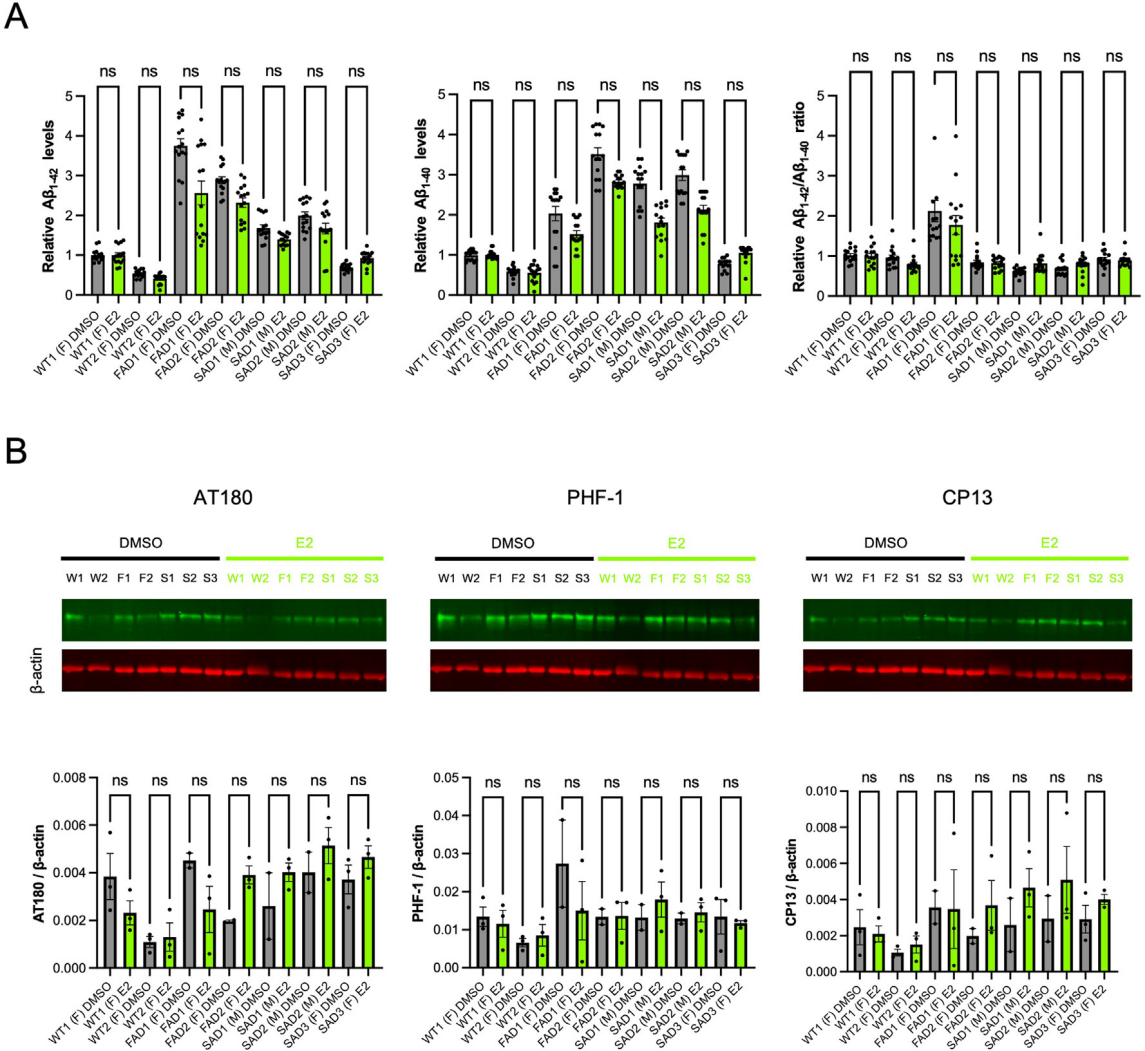
The effects of E2 treatment on AD pathologies of the iPSC-derived neurons. **A** Treatment with 100 nM E2 for 2 months to the iPSC-derived neurons at PID 45 did not change the levels of the secreted amyloid-beta (Aβ_1-42_, Aβ_1-40_, and Aβ_1-42_/Aβ_1-40_ ratio) in every cell line (n = 15 each). ns: non-significant (Kruskal-Wallis test). **B** Treatment with 100 nM E2 for 2 months to the iPSC-derived neurons at PID 45 did not change the level of phosphorylated Tau proteins (AT180, PHF-1, and CP13) in every cell line (n = 3 each). ns: non-significant (Kruskal-Wallis test).

The E2 treatment was shown to alleviate the AD pathogenesis including Aβ and pTau in the previously reported study using an animal model of AD [11]. Therefore, we aimed to investigate the E2 effects on the AD pathogenesis in our induced neurons from the human iPSCs. To make the length of treatment comparable to that in the mouse study, the induced neurons were treated with 100 nM E2 for 2 months before the measurement of secreted Aβ and pTau. As a result, the long-term E2 treatment reduced neither the secreted Aβ in the conditioned media nor the levels of pTau in the cell lysates compared to the vehicle treatment in the induced neurons. The same results were obtained in all the neurons derived from every iPSC line used in our study regardless of sex and the AD/non-AD status (Fig. 5A and B). Thus, even 2 months of E2 treatment did not reduce the levels of Aβ and phosphorylated Tau in the present iPSC-derived neuronal models.

### 3.5 The co-culture model of neurons and astrocytes induced from familial AD patient's iPSCs expressed astrogliosis-like phenotype and E2 treatment alleviated the phenotype

**Fig. 6:**
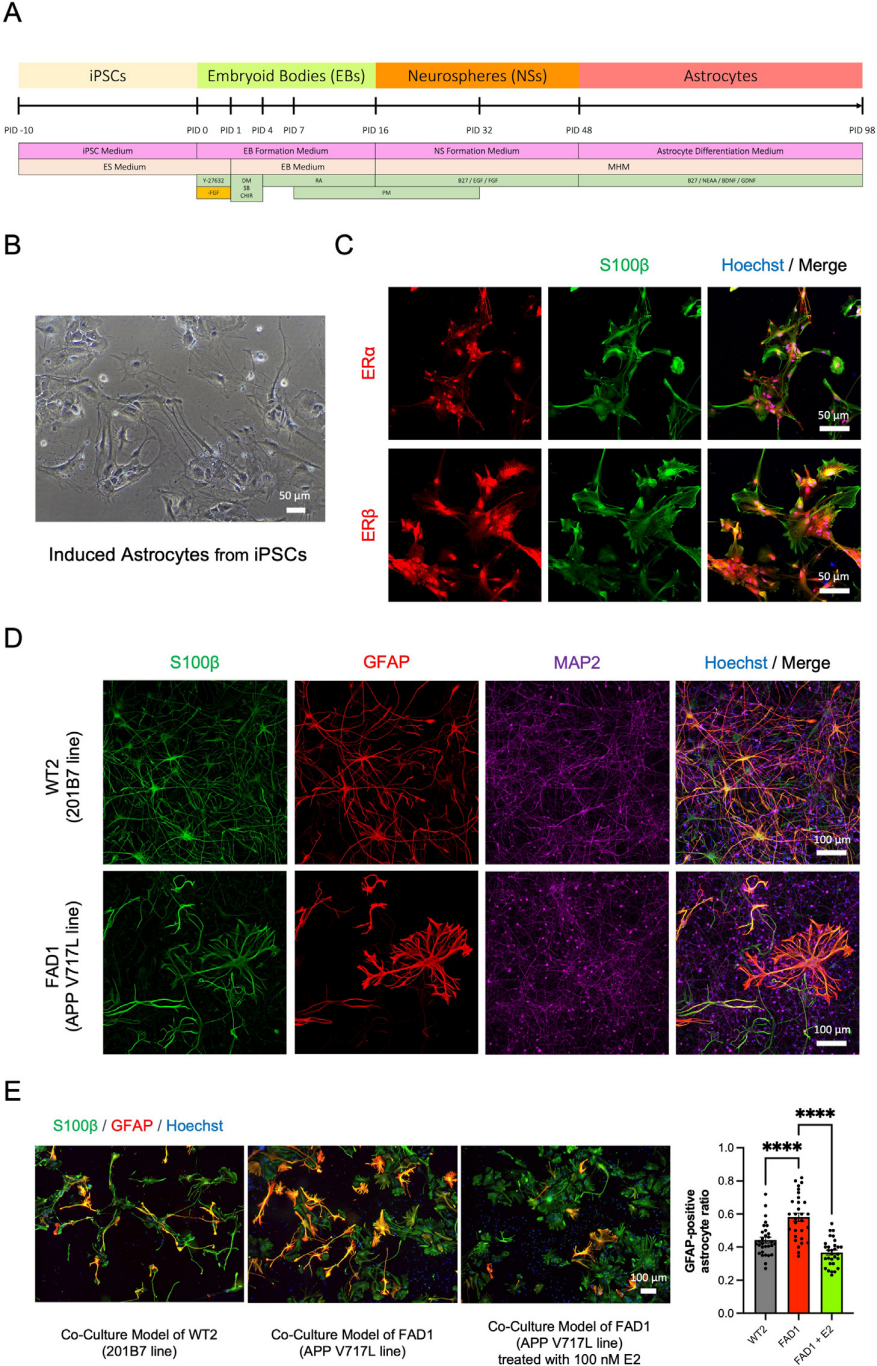
Treatment with E2 alleviated the astrogliosis-like phenotype developed in the fAD iPSC-derived co-culture model. **A** Induction method for the differentiation of astrocytes from iPSCs. **B** Bright-field image of human astrocytes induced from iPSCs. Scale bar; 50 μm. **C** Immunocytochemistry images of iPSC-derived astrocytes after PID 98 stained with markers of estrogen receptors (ERs) (ERα and ERβ). Scale bar; 50 μm. **D** Immunocytochemistry images of the co-culture model derived from the control (WT2 line) and the AD (FAD1 line) donors at 30 days after co-culturing the iPSC-derived neurons and astrocytes stained with S100β, GFAP, and MAP2. Scale bar; 100 μm. **E** Treatment with 100 nM E2 for 4 days alleviated the astrogliosis-like phenotype in the co-culture model generated from AD donor (FAD1 line) (n = 30 each). Scale bar; 100 μm. Bars, mean ± SEM. ∗∗∗∗*p* < 0.0001 (One-way ANOVA).

To evaluate the effects of E2 treatment in the mixture of different cell types that is more relevant to the condition in brains, we utilized the newly established co-culture model of neurons and astrocytes induced from the identical iPSCs [28]. The astrocytes were induced from the iPSCs with the previously reported protocol from our group (Fig. 6A and B) [27]. To confirm if the induced astrocytes are capable of responding to E2 treatment, we confirmed that the induced astrocytes expressed the estrogen receptors (Fig. 6C). Then, we co-cultured the neurons and astrocytes and confirmed that both neurons and astrocytes survived even 30 days after co-culturing with neuronal culture medium (Fig. 6D).

Next, to assess the E2 treatment effects on astrocytic AD phenotypes, we also generated the co-culture model from the familial AD (APP V717L) (FAD1) iPSC line (Fig. 6D). The ratio of GFAP/S100β-positive cells of the fAD line was higher than that of the healthy control (WT2) iPSC line (Fig. 6E). Thus, this suggested the astrogliosis-like phenotype that can be observed using the co-culture model of fAD patient (APP V717L). In addition, the treatment with 100 nM E2 for 4 days ameliorated the astrogliosis-like phenotype of the co-culture from the fAD line [28] (Fig. 6E).

## 4 Discussion

Estrogen, particularly estradiol (E2), is a female sex hormone associated with the development and regulation of the female reproductive system and secondary sexual characteristics. Additionally, this hormone has been reported to exhibit protective effects on the central nervous system. E2 treatment has shown multiple positive effects in improving the pathogenesis of the AD mouse model [3,8]. For instance, E2 administration has been observed to reduce the formation of Aβ plaques, subsequently ameliorating symptoms in mouse models with the AD mutation [11,51]. One study suggested that E2 reduces Aβ plaque formation by elevating Aβ protease activity through the leptin/IGF-1/somatostatin signaling pathway [52]. Using the forebrain-neuron-specific aromatase knock-out (FBN-ARO-KO) mouse model, another study highlighted that exogenous E2 administration ameliorated the reduction of spines and synaptic densities in forebrains. Moreover, E2 administration rescued the long-term potentiation (LTP) defect in this model, underscoring the role of E2 in promoting synaptic plasticity [53]. However, in our study, E2 did not enhance synapses or dendritic spines, which may be attributed to differences between human and mouse cells. Additionally, E2 is effective in controlling inflammation by regulating glial cells (Reviewed in [54]). In our co-culture model of human iPSCs-derived neurons and astrocytes, the astrogliosis-like phenotype shown in fAD line, APP V717L [28], was alleviated by E2 treatment. This suggests that in both humans and mice, astrocytes, rather than neurons, share common pathways downstream of E2.

However, at the human level, it remains unclear whether E2 could prevent AD and is effective against AD pathogenesis. Some clinical trials showed favorable effects of estrogen replacement therapy in postmenopausal AD patients, preserving or promoting cognitive function [55], improving metabolism in the brain [56,57], and enhancing mood [58,59]. Nevertheless, some studies concluded that estrogen has no significant effects on preventing and improving AD symptoms [12,[60], [61], [62]] (Supplementary Table 1). Our meta-analysis suggested that estrogen replacement therapy is likely to be effective in preventing AD, consistent with another meta-analysis study [63]. Recent randomized clinical trials concluded that estrogen promotes cognitive functions in certain conditions (females with fewer years of menopausal or the APOE ε4 genotype). The type of administration (transdermal vs oral) is also associated with the treatment effectiveness [56,57,59,64].

Utilizing the cellular models from the human iPSCs, this study demonstrated the positive effects of E2 on human neuronal cells. However, we could not find a significant effect of E2 in some readouts that originally expected to see significant effects from previous reports using murine neurons. Especially, the effects on promoting the neuronal activities observed with the increased Ca^2+^ oscillations after short-term E2 treatment and the enhancement of neurite branching after long-term E2 treatment were reminiscent of the previous studies using the models of human embryonic stem cells (ESCs) and iPSCs [49,50]. Unlike these studies that included only healthy control iPSC lines, we evaluated the E2 effects on several iPSC lines derived from healthy and AD donors of males and females. Then, we found that E2 treatment was effective in promoting neurite extension and neuronal activity regardless of sex and the AD/non-AD status.

E2 treatment showed no effects on the hallmark AD pathology including the Aβ secretion and pTau accumulation in the mono-culture model. Some previous reports demonstrated that E2 is effective in reducing Aβ secretions from human embryonic cerebrocortical neurons in a dose-dependent manner [65], and E2 could reduce the Aβ accumulation in the cortical brain regions of an ovariectomized 3xTg AD mouse model [11]. For the effects of E2 on tau proteins, previous reports suggested that E2 is effective in reducing pTau via the inhibition of glycogen synthase kinase-3beta (GSK-3β) and is effective specifically on the hyperphosphorylation at Ser396/404, Ser202/Thr205, and Ser199/202 [11,[66], [67], [68]]. However, the long-term E2 treatment on human neuronal models showed no changes in Aβ secretion and pTau accumulation, which were different from the previously reported results in the animal models, also indicating the difference between human and mouse neurons.

In the co-culture model comprising neurons and astrocytes, E2 could alleviate the astrogliosis-like phenotypes likely caused by the elevated Aβ secretion in the fAD iPS line. Some of the previous studies have shown that E2 downregulated the number of GFAP-positive astrocytes in the rat models under pathological conditions [[69], [70], [71]]. Another recent study using the mouse model of global cerebral ischemia also demonstrated that E2 administration ameliorated reactive astrogliosis [72]. E2 treatment can target directly astrocytes because estrogen receptors are also expressed in astrocytes. Thus, these findings suggested the protective effects of estrogen directly on the reactive human astrocytes under pathological conditions like previously shown in rat astrocytes [73]. E2 treatment could not reduce the secreted Aβ levels in our neuronal mono-culture model. However, E2 treatment reduced the amount of Aβ plaque in the ovariectomized AD mouse model [11]. The E2 effects on Aβ in the mouse models could be due to the enhancement of non-neuronal cells that were not examined in this study. By utilizing the co-culture model of neurons and glial cells derived from iPSCs, which could enhance the cellular maturation and astrocytic phenotype of a fAD iPSC [28], further investigation into the molecular mechanisms of E2 effects on AD pathogenesis will be required.

Lastly, there are several limitations of this study that need to be addressed. First, our neural models were induced from the iPSCs, which are the rejuvenated cells. As AD is a neurodegenerative disease developing in old age, the juvenile characteristics of the neural models might prevent the complete recapitulation of the old neurons. The effects of the E2 treatment observed in the mouse model could have been hindered due to the juvenile characteristics of the differentiated cells. Second, although our work demonstrated some of the E2 treatment effects on the human cells with hormone-free status, the models might not fully recapitulate the menopausal stage where the neurons were exposed to the hormone for a long period of time before the depletion of the hormone. Generation of the menopausal model in human iPSCs-derived models, in which the models are once exposed to E2 and then deplete the E2 to mimic the menopause condition, is suggested. Such a transition to a hormone-free condition might better recapitulate the pathogenesis of AD in women undergoing menopause. Then, the re-exposure to E2 in these models could demonstrate the effects of E2 in menopaused AD women and the results can be compared to the clinical trials as aforementioned. In addition, a recent study suggested that the follicle-stimulating hormone (FSH), another hormone that reversely correlates with E2, promoted the deposition of Aβ and Tau both *in vitro* and *in vivo* [74]. Therefore, the exposure of iPSC-derived cells to FSH may further elicit pathological changes of Aβ and Tau [75] in our models, and then in the elicited condition, E2 may attenuate the levels of secreted Aβ or pTau accumulation.

## 5 Conclusion

This study demonstrated the effects of estradiol (E2) treatment on human cellular models derived from the iPSCs irrespective of the donor's sex or disease status. The induced neurons and astrocytes were found to express estrogen receptors and exhibited both acute and chronic responses to E2 treatment. This was evident from the increased Ca^2+^ oscillations and the promotion of neurite branching. While E2 did not modify the changes in secreted Aβ levels or pTau levels in the neuronal model, the treatment did mitigate the astrogliosis-like phenotype observed in the co-culture model of neurons and astrocytes.

## Authors contributions

SS: conceptualization, study design, conducting experiments, data analysis, data interpretation, visualization, and original manuscript preparation. CO: conducting experiments, data analysis, and visualization. HY: conducting experiments, data analysis, and visualization. SM: conceptualization, study design, data interpretation, writing, and editing the manuscript. HO: data interpretation, reviewing, and editing the manuscript. All authors have read and approved the manuscript.

## Funding

This research was funded by the Grants-in-Aid for Scientific Research (KAKENHI, #21J21244 to SS, #21H02450, #23K18116, and #21K06376 to SM), the Keio Global Research Institute from Keio University (to SM and HO), the Japan Agency for Medical Research and Development (AMED) [The Acceleration Program for Intractable Disease Research Utilizing Disease-specific iPS Cells to SM and HO (JP21bm0804003)], [Research and Development Grants for Dementia to SM (22dk0207060)], the Keio University Doctorate Student Grant-in-Aid Program from Ushioda Memorial Fund 2021 (to SS), and the research grant from Keio Medical Association (the fiscal year 2022) (to SS).

## Data availability statement

The original contributions presented in this study are included in the article/Supplementary material, further inquiries are available from the corresponding authors upon request.

## Ethics statement

The study was conducted per the Declaration of Helsinki, approved by the Ethics Committee for Human Research of the Keio University School of Medicine (#20080016).

## Declaration of competing interest

Hideyuki Okano is a founder scientist and a Scientific Advisory Board Member for SanBio Co., Ltd., and K Pharma Inc. The remaining authors declare no conflicts of interest.
