# Supplementary material for: Estradiol enhanced neuronal plasticity and ameliorated astrogliosis in human iPSC-derived neural models
Source: Regen Ther. 2024 Jan 12;25:250–63. doi: 10.1016/j.reth.2023.12.018 (PMC10826128; doi:10.1016/j.reth.2023.12.018)
Supplement: Multimedia component 1 [file mmc1.docx]

**Estradiol enhanced neuronal plasticity and ameliorated astrogliosis in human iPSC-derived neural models**

Sopak Supakul ^1^, Chisato Oyama ^2^, Yuki Hatakeyama ^1^, Sumihiro Maeda ^1,*^, Hideyuki Okano ^1,*^

^1^ Department of Physiology, Keio University School of Medicine, Tokyo, Japan

^2^ Department of Electrical Engineering and Bioscience, School of Advanced Science and Engineering, Waseda University, Tokyo, Japan

^*^ Correspondence: sumihiro.maeda@keio.jp (S.M.) and hidokano@a2.keio.jp (H.O.)

**
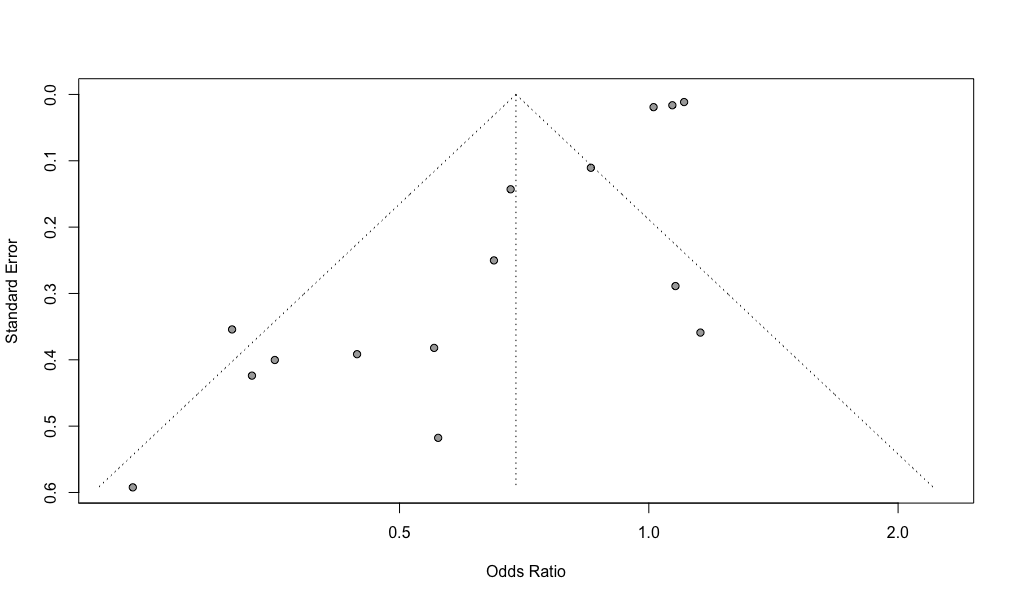
**

**Supplementary Fig 1.** Funnel plot of the studies included in the meta-analysis.

**Supplementary Table 1.** Summary of the results on estrogen replacement therapy and AD risk of studies included in the meta-analysis.

| No. | Author, Year | Study design | Mean Age (y.o.) | Diagnosis Creteria | AD | | | Control | | | OR/AR/RR | 95% CI | Main Results |
| --- | --- | --- | --- | --- | --- | --- | --- | --- | --- | --- | --- | --- | --- |
|  |  |  |  |  | All HRT | Estrogen only | Control | All HRT | Estrogen only | Control |  |  |  |
| 1 | Henderson et al. 1994 | Case-control study | 76.1 | Neurological history; Neurological, neuro-psychologic, and laboratory assessments | 10 | 10 | 133 | 17 | 17 | 75 | − | − | ERT is associated with a reduced risk of AD in postmenopausal women. |
|  |  |  |  |  |  |  |  |  |  |  |  |  |  |
| 2 | Mortel et al. 1995 | Case-control study | 72.8 | NINCDS-ADRDA | 11 | 11 | 82 | 29 | 29 | 119 | 1.82 | 0.859-3.842 | Lack of ERT is associated with increased risk of AD. |
|  |  |  |  |  |  |  |  |  |  |  |  |  |  |
| 3 | Tang et al. 1996 | Prospective cohort study | 74.2 | NINCDS-ADRDA | 9 | 9 | 158 | 147 | 147 | 810 | 0.4 | 0.22-0.85 | ERT is associated with a reduced risk of AD in postmenopausal women. |
|  |  |  |  |  |  |  |  |  |  |  |  |  |  |
| 4 | Paganini-Hill et al. 1996 | Nested case-control study | 87.3 | Medical records; Diagnosis of AD listed on the death certificate | 96 | 96 | 150 | 578 | 578 | 615 | 0.65 | 0.49-0.88 | The risk of AD and related dementia was significantly reduced in estrogen users compared with non-users. |
|  |  |  |  |  |  |  |  |  |  |  |  |  |  |
| 5 | Kawas et al. 1997 | Prospective cohort study | 61.5 | NINCDS-ADRDA | 9 | 9 | 25 | 221 | 221 | 217 | 0.46 | 0.209-0.997 | Women who had reported the use of estrogen had a reduced risk of AD. |
|  |  |  |  |  |  |  |  |  |  |  |  |  |  |
| 6 | Baldereschi et al. 1998 | Prospective cohort study | 74.5 | NINCDS-ADRDA | 3 | 3 | 89 | 183 | 183 | 1293 | 0.24 | 0.07-0.77 | ERT is associated with a reduced prevalence of AD in postmenopausal women. |
|  |  |  |  |  |  |  |  |  |  |  |  |  |  |
| 8 | Slooter et al. 1999 | Case-control study | 58.1 | NINCDS-ADRDA | 11 | 11 | 98 | 24 | 24 | 95 | 0.34 | 0.12-0.94 | Estrogen use is beneficial to AD with early onset. |
|  |  |  |  |  |  |  |  |  |  |  |  |  |  |
| 7 | Waring et al. 1999 | Case-control study | 82 | NINCDS-ADRDA | 33 | 33 | 189 | 47 | 47 | 175 | 0.42 | 0.18-0.96 | ERT is associated with a reduced risk of AD in postmenopausal women. |
|  |  |  |  |  |  |  |  |  |  |  |  |  |  |
| 9 | Seshadri et al. 2001 | Nested case-control study | 65.5 | NINCDS-ADRDA | 15 | 4 | 44 | 53 | 16 | 168 | Estrogen only: 0.89 Estrogen + Progestin: 1.45 | Estrogen only: 0.35-2.30 Estrogen + Progestin: 0.60-3.49 | HRT is not associated with reduced risk of developing AD. |
|  |  |  |  |  |  |  |  |  |  |  |  |  |  |
| 10 | Lindsay et al. 2002 | Prospective cohort study | 82 | NINCDS-ADRDA | 4 | 4 | 106 | 125 | 125 | 1844 | 1.37 | 0.48-3.95 | Estrogen was not shown to be protective against development of AD. |
|  |  |  |  |  |  |  |  |  |  |  |  |  |  |
| 11 | Roberts et al. 2006 | Case-control study | 84 | DSM-IV | 28 | 28 | 216 | 26 | 26 | 216 | 1.1 | 0.63-1.93 | No significant difference between those who received HRT and the control. |
|  |  |  |  |  |  |  |  |  |  |  |  |  |  |
| 12 | Imtiaz et al. 2017 | Prospective cohort study | 52.1 | NINCDS-ADRDA; DSM-IV | 68 | 68 | 209 | 2230 | 2230 | 5688 | 0.92 | 0.68-1.20 | ERT is associated with a reduced risk of AD in postmenopausal women. |
|  |  |  |  |  |  |  |  |  |  |  |  |  |  |
| 13 | Imtiaz et al. 2017 | Case-control study | 63.9 | NINCDS-ADRDA; DSM-IV | 9127 | 3848 | 36990 | 33951 | 14753 | 150512 | Estrogen: 1.10 Progesterone: 1.13 | Estrogen: 1.06-1.12 Progesterone: 1.10-1.17 | Only a use of systemic estrogen HT for >10 years (OR, 95% CI: 0.91, 0.84–0.99) was protective against AD. |
|  |  |  |  |  |  |  |  |  |  |  |  |  |  |
| 14 | Tolppanen et al. 2018 | Nested case-control study | 74 | NINCDS-ADRDA; DSM-Ⅳ | 6352 | 5206 | 39764 | 6280 | 5158 | 39836 | AD cohort: 0.68. Control cohort: 0.71 | AD cohort: 0.55-0.84 Control cohort: 0.59-0.85 | ERT is associated with a reduced risk of AD in postmenopausal women. |
|  |  |  |  |  |  |  |  |  |  |  |  |  |  |
| 15 | Savolainen-Peltonen et al. 2019 | Case-control study | 57.9 | NINCDS-ADRDA; DSM-Ⅳ | 26553 | 5606 | 58186 | 25564 | 5312 | 59175 | Estrogen only: 1.09 Estrogen + Progestin:1.17 | Estrogen only: 1.05-1.14 Estrogen + Progestin: 1.13-1.21 | ERT is not associated with a reduced risk of AD in postmenopausal women. |

AD: Alzheimer's Disease; DSM-Ⅳ: Diagnostic and Statistical Manual of Mental Disorders Fourth Edition; HRT: Hormone Replacement Therapy; NINCDS-ADRDA: National Institute of Neurological and Communicative Diseases and Stroke and Alzheimer Disease and Related Disorders Association.

**Supplementary Table 2.** Antibodies and primers used in this study.

| **Antibodies used for immunocytochemistry and western blot** | | | |
| --- | --- | --- | --- |
|  | **Antibody** | **Dilution** | **Company Cat # and RRID** |
| Neuronal marker (ICC) | Mouse Anti-MAP2 | 1:100 | SIGMA Cat# M4403  RRID:AB_477193 |
| Neuronal marker (ICC) | Rabbit Anti-MAP2 | 1:100 | Cell Signaling Technology Cat# 4542 RRID:AB_10693782 |
| Neuronal marker (ICC) | Mouse Anti-NeuN | 1:200 | Abcam Cat# ab104224  RRID:AB_10711040 |
| Neuronal marker (ICC) | Rat Anti-Tau (RTM38) | 1:2000 | Wako Cat# 017-26893 |
| Synaptic marker (ICC) | Rabbit Anti-Synapsin1 | 1:2000 | Sigma-Aldrich Cat# S193  RRID:AB_261457 |
| Estrogen receptor  marker (ICC) | Rabbit Anti-Estrogen Receptor α | 1:2000 | Abcam Cat# ab3575  RRID: AB_303921 |
| Estrogen receptor  marker (ICC) | Rabbit Anti-Estrogen Receptor β | 1:100 | Abcam Cat# ab3576  RRID: AB_303922 |
| Fluorescence protein marker (ICC) | Rabbit Anti-RFP | 1:500 | MBL International Cat# PM005  RRID:AB_591279 |
| Astrocyte marker (ICC) | Rat Anti-GFAP | 1:500 | Millipore Cat# 345860  RRID:AB_2109651 |
| Astrocyte marker (ICC) | Mouse Anti-S100β | 1:250 | Sigma-Aldrich Cat# S2532 RRID:AB_477499 |
| Phosphorylated tau marker (WB) | Mouse Anti-Phospho-tau  (Thr231) (AT180) | 1:500 | Thermo Fisher Scientific Cat# MN1040 RRID:AB_223649 |
| Phosphorylated tau marker (WB) | Mouse Anti-Phospho-tau (Ser396/Ser404) (PHF-1) | 1:1000 | Peter Davies Laboratory |
| Phosphorylated tau marker (WB) | Mouse Anti-Phospho-tau (Ser202) (CP13) | 1:1000 | Peter Davies Laboratory |
| Secondary Antibody | Alexa Flour 488  Goat Anti-Rabbit IgG | 1:500 | Thermo Fisher Scientific Cat# A11034 RRID:AB_2576217 |
| Secondary Antibody | Alexa Flour 555  Goat Anti-Rabbit IgG | 1:500 | Molecular Probes Cat# A21429  RRID:AB 2535850 |
| Secondary Antibody | Alexa Flour 647  Goat Anti-Rabbit IgG | 1:500 | Molecular Probes Cat# A21245 RRID:AB_141775 |
| Secondary Antibody | Alexa Flour 488  Goat Anti-Mouse IgG | 1:500 | Molecular Probes Cat# A11029  RRID:AB_2534088 |
| Secondary Antibody | Alexa Flour 555  Goat Anti-Mouse IgG | 1:500 | Molecular Probes Cat# A21424  RRID:AB_141780 |
| Secondary Antibody | Alexa Flour 647  Goat Anti-Mouse IgG | 1:500 | Thermo Fisher Scientific Cat# A21236 RRID:AB_2535805 |
| Secondary Antibody | Alexa Flour 555  Goat Anti-Rat IgG | 1:500 | Molecular Probes Cat# A21434  RRID:AB_141733 |
| Secondary Antibody | Alexa Flour 647  Goat Anti-Rat IgG | 1:500 | Molecular Probes Cat# A21247  RRID:AB_141778 |
| **Primers** | | | |
|  | **Target/Product length** | **Forward/Reverse primer (5′-3′)** | |
| Differentiation marker (qPCR) | *OCT4*/144bp | F: GACAGGGGGAGGGGAGGAGCTAG R: CTTCCCTCCAACCAGTTGCCCCA | |
| Differentiation marker (qPCR) | *SOX2*/151bp | F: GGGAAATGGGAGGGGTGCAAAAG  R: TTGCGTGAGTGTGGATGGGATTG | |
| Neuronal marker (qPCR) | *FOXG1*/131bp | F: CCCGTCAA TGACTTCGCAGA  R: GTCCCGTCGTAAAACTTGGC | |
| Neuronal marker (qPCR) | *PAX6*/109bp | F: ACCACACCGGTTTCCTCCTTCACA  R: TTGCCATGGTGAAGCTGGGCAT | |
| House-Keeping Gene | *ACTB*/151bp | F: TGAAGTGTGACGTGGACATC   R: GGAGGAGCAATGATCTTGAT | |

**Supplementary Fig 2.** Tracing of neurite by the sparse-labeling of iPSC-derived neurons at PID 45 using the pCl-DsRed. **A** Structure of pCl-dsRed vector for the sparse-labeling of neurons. **B** Immunocytochemistry (RFP and MAP2) images of WT1 iPSC-derived neurons (1210B2 line) after transfection with pCl-dsRed and 4-day treatment with 100 nM E2. Scale bar; 50 μm.

**Supplementary Fig 3.** Treatment with 17β-estradiol (E2) resulted in increased neuronal activities and neurite branching of the iPSC-derived neurons after PID 45 (line WT2, FAD2, SAD1, and SAD2). **A** Ca^2+^ oscillations of the iPSC-derived neurons at PID 45 after 15-min 100 nM E2 treatment measured with Ca^2+^ imaging (n = 45 each). WT2: **p* = 0.0116 & ***p* = 0.0087; FAD2: ns (non-significant) & *****p* < 0.0001; SAD1: *****p* < 0.0001 & *****p* < 0.0001; SAD2: ns (non-significant) & *****p* < 0.0001 (Mann Whitney test). **B** Increased neurite branching of iPSC-derived neurons after 4-day 100 nM E2 treatment measured by Sholl analysis (n = 20 each). WT2, FAD2, SAD1, SAD2: *****p* < 0.0001 (Two-way ANOVA).

**Supplementary Fig 4.** The effects of E2 treatment on changes in the number of synapses in WT1 iPSC-derived neurons. **A** Immunocytochemistry image of iPSC-derived neurons treated with 100 nM E2 for 4 days stained with Synapsin I and MAP2. Scale bar; 20 μm. **B** Quantification of Synapsin I expression on the neurite of iPSC-derived neurons (Number of Synapsin I / μm) between DMSO treatment group (n = 20) and E2 treatment group (n = 20) in all the cell lines. ns: non-significant (Mann-Whitney test).

**Supplementary Fig 5.** The effects of E2 treatment on changes in the number of dendritic spines in WT1 iPSC-derived neurons. **A** Immunocytochemistry image of iPSC-derived neurons at PID 45 transfected with β-actin-EGFP and stained with anti GFP antibodies. Scale bar; 20 μm. **B** Different types of dendritic spines appeared on the iPSC-derived neurons at PID 45. **C** Quantification of the total number of dendritic spines of iPSC-derived neurons (WT1) (Number of spines / μm) between DMSO treatment group (n = 10) and E2 treatment group (n = 10). ns: non-significant (Mann-Whitney test). **D** Quantification of the number of dendritic spines by types of the iPSC-derived neurons (Number of spines / μm) between DMSO treatment group (n = 10) and E2 treatment group (n = 10). ns: non-significant (Kruskal-Wallis test). **E** Quantification of the total number of dendritic spines of iPSC-derived neurons (Number of spines / μm) between DMSO treatment group (n = 20) and E2 treatment group (n = 20) in all the cell lines. ns: non-significant (Kruskal-Wallis test).
