# Supplementary material for: Estradiol enhanced neuronal plasticity and ameliorated astrogliosis in human iPSC-derived neural models
Source: Regen Ther. 2024 Jan 12;25:250–63. doi: 10.1016/j.reth.2023.12.018 (PMC10826128; doi:10.1016/j.reth.2023.12.018)
Supplement: Multimedia component 2 [file mmc2.pptx]

## Slide 1
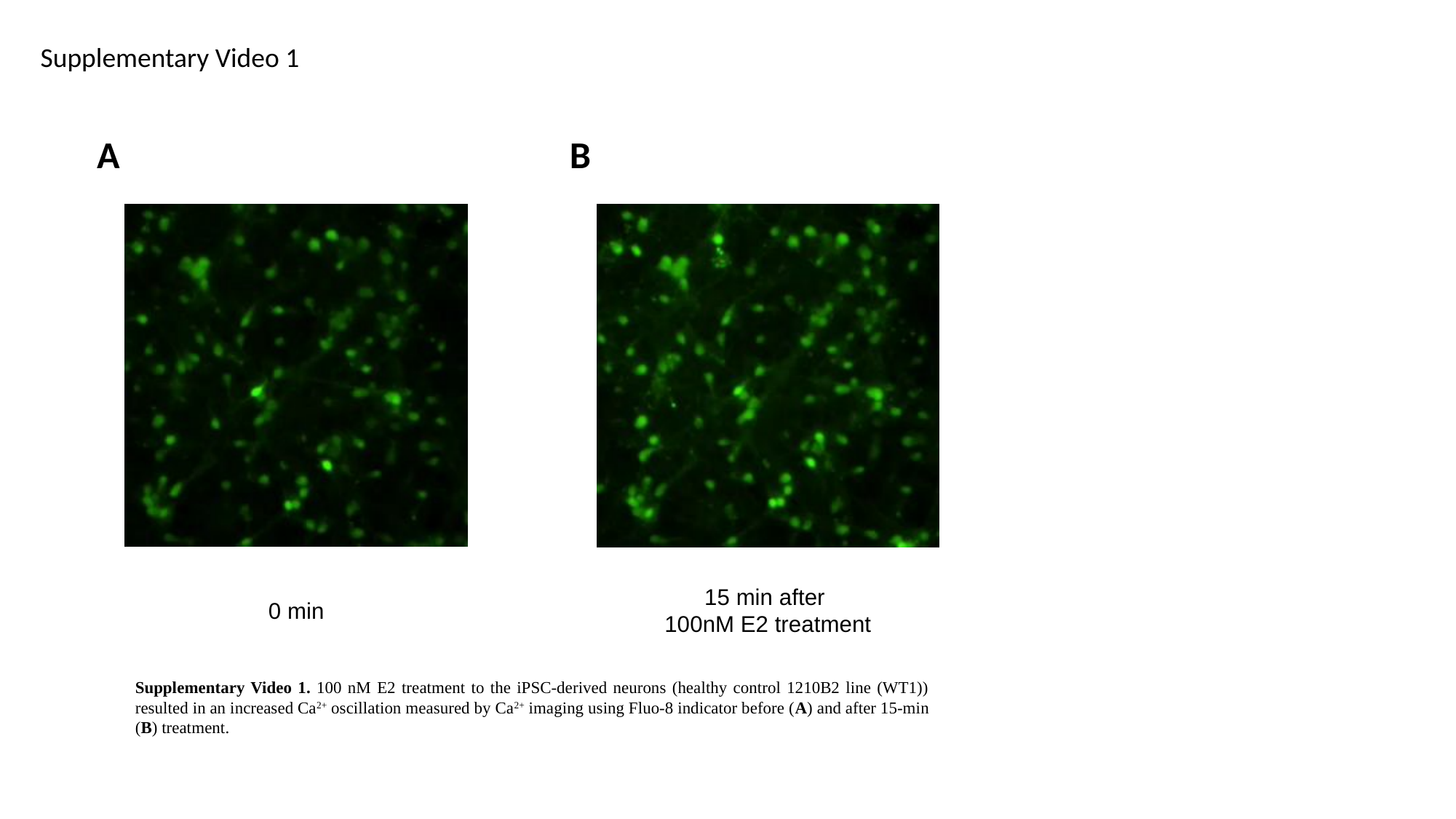

Supplementary Video 1
A
B
15 min after
100nM E2 treatment
0 min
Supplementary Video 1. 100 nM E2 treatment to the iPSC-derived neurons (healthy control 1210B2 line (WT1)) resulted in an increased Ca2+ oscillation measured by Ca2+ imaging using Fluo-8 indicator before (A) and after 15-min (B) treatment.
